# Effects of diaphragmatic contraction on lower limb venous return and central hemodynamic parameters contrasting healthy subjects versus heart failure patients at rest and during exercise

**DOI:** 10.14814/phy2.12216

**Published:** 2014-12-11

**Authors:** Fernanda Machado Balzan, Régis Chiarelli da Silva, Danton Pereira da Silva, Paulo Roberto Stefani Sanches, Angela Maria Vicente Tavares, Jorge Pinto Ribeiro, Danilo Cortozi Berton, Nadine Oliveira Clausell

**Affiliations:** 1 Exercise Pathophysiology Research Laboratory, Programa de Pós‐Graduação em Ciências da Saúde, Cardiologia e Ciências Cardiovasculares, Universidade Federal do Rio Grande do Sul (UFRGS), Hospital de Clinicas de Porto Alegre (HCPA), Porto Alegre, RS, Brazil; 2 Hospital Santa Casa de Misericórdia de Porto Alegre, Porto Alegre, RS, Brazil; 3 Biomedical Engineering Division, Universidade Federal do Rio Grande do Sul (UFRGS), Hospital de Clinicas de Porto Alegre (HCPA), Porto Alegre, RS, Brazil; 4 Faculdade de Ciências da Saúde Uniritter ‐ Centro Universitário Ritter dos Reis, Porto Alegre, RS, Brazil; 5 Respiratory Division, Programa de Pós‐Graduação em Ciências Pneumológicas, Universidade Federal do Rio Grande do Sul (UFRGS), Hospital de Clinicas de Porto Alegre (HCPA), Porto Alegre, RS, Brazil; 6 Cardiology Division, Programa de Pós‐Graduação em Ciências da Saúde, Cardiologia e Ciências Cardiovasculares, Universidade Federal do Rio Grande do Sul (UFRGS), Hospital de Clinicas de Porto Alegre (HCPA), Porto Alegre, RS, Brazil

**Keywords:** Cardiac output, diaphragmatic contraction, venous return

## Abstract

The main objective was to assess the effects of abdominal breathing (AB) versus subject's own breathing on femoral venous blood flow (*Q*_fv_) and their repercussions on central hemodynamics at rest and during exercise contrasting healthy subjects versus heart failure (HF) patients. We measured esophageal and gastric pressure (*P*_GA_), *Q*_fv_ and parameters of central hemodynamics in eight healthy subjects and nine HF patients, under four conditions: subject's own breathing and AB (**∆***P*_GA_ ≥ 6 cmH_2_O) at rest and during knee extension exercises (15% of 1 repetition maximum) until exhaustion. *Q*_fv_ and parameters of central hemodynamics [stroke volume (SV), cardiac output (CO)] were measured using Doppler ultrasound and impedance cardiography, respectively. At rest, healthy subjects *Q*_fv_, SV, and CO were higher during AB than subject's breathing (0.11 ± 0.02 vs. 0.06 ± 0.00 L·min^−1^, 58.7 ± 3.4 vs. 50.1 ± 4.1 mL and 4.4 ± 0.2 vs. 3.8 ± 0.1 L·min^−1^, respectively, *P* ≤ 0.05). ∆SV correlated with ∆*P*_GA_ during AB (*r* = 0.89, *P* ≤ 0.05). This same pattern of findings induced by AB was observed during exercise (SV: 71.1 ± 4.1 vs. 65.5 ± 4.1 mL and CO: 6.3 ± 0.4 vs. 5.2 ± 0.4 L·min^−1^; *P* ≤ 0.05); however, *Q*_fv_ did not reach statistical significance. The HF group tended to increase their *Q*_fv_ during AB (0.09 ± 0.01 vs. 0.07 ± 0.03 L·min^−1^, *P *= 0.09). On the other hand, unlike the healthy subjects, AB did not improve SV or CO neither at rest nor during exercise (*P *> 0.05). In healthy subjects, abdominal pump modulated venous return improved SV and CO at rest and during exercise. In HF patients, with elevated right atrial and vena caval system pressures, these findings were not observed.

## Abstract (precis)

Circulatory function of the diaphragm produces an increase in circulatory output. Moreover, the peripheral muscle contraction produces greater venous blood return due to increased blood expulsion. In this study, we focused on the effects of diaphragm contraction at rest and during knee extension exercise on venous return and central hemodynamics in healthy subjects and heart failure patients. These results help us understand the mechanisms of abdominal pump modulation on venous return in healthy subjects and under conditions of elevated pressure of the right atrium and the vena cava.

## Introduction

There are contradictory results in the literature regarding the effects of inspiratory diaphragmatic descent (increase in abdominal pressure; *P*_AB_) on venous return. During quiet breathing, beneficial hemodynamic effects of diaphragm contractions induced by phrenic pacing have been described in dogs (Ishii et al. 1990) and in humans (Roos et al. 2009). However, others have demonstrated that femoral blood flow (*Q*_fv_) decreased more during diaphragm contractions and as a result, contributed more to inspiration with a greater increase in *P*_AB_ (Willeput et al. 1984). During “pure” diaphragmatic breathing, blood flow completely stopped, whereas during predominant rib cage inspiration, the blood flow increased. Isovolume belly‐in maneuvers and gentle external compression of the abdomen also caused cessation of *Q*_fv_, indicating that *P*_AB_ rather than diaphragmatic contraction is the mechanism which explains why the venous return from the legs is impeded during inspiration (Miller et al. 2005a).

More recently, it has been demonstrated (Aliverti et al. 2009, 2010) that the modulation of the splanchnic vascular bed as a result of an increase in *P*_AB_ via diaphragmatic contraction may contribute to inferior vena caval blood return, that is, during inspiration, splanchnic venous return is favored, whereas, during expiration, a venous return of *Q*_fv_ below the entry of the hepatic vein is favored. Thus, a greater diaphragm contraction contributed to abdominal circulatory pump results in hemodynamic benefits secondary to net increase in blood venous return and, through Frank‐Starling mechanism (Cingolani et al. 2013), greater cardiac stroke volume (SV) (Aliverti et al. 2010). To corroborate all this rational, we aimed to contrast healthy subjects against heart failure (HF) patients. We believe that the impact of diaphragmatic contractions on venous blood return and central hemodynamics would be lower in HF patients who presented overloaded cardiac chambers and limited Frank‐Starling reserve.

Additionally, during dynamic exercise, the contraction of peripheral skeletal muscles causes compression of the intramuscular veins, furthermore, facilitating the return of the blood to the heart (Hogan et al. 2003; Stewart et al. 2004). Accordingly, during exercise, the addition of peripheral locomotor limb muscular contraction could affect the respiratory modulation of venous return from the lower limbs. Therefore, we also aimed to investigate the impact of modulating diaphragmatic contractions on venous blood return and central hemodynamics comparing healthy and heart failure subjects during dynamic exercise. With the goal of finding out whether modulation in breathing pattern could be beneficial in terms of physiological as well as clinical responses (effort perception and exercise tolerance) during exercise in healthy subjects. Again, we believe that no positive (or even detrimental) effects would be seen in HF patients.

## Methods

### Subjects

Clinically stable, male patients with chronic HF (New York Heart Association [NYHA] functional class I & II) were recruited from the Hospital de Clínicas de Porto Alegre between January and December 2012. Matched male volunteers with no history of cardiac or respiratory disease were recruited as healthy controls. Prior to participation in the study, all subjects were informed of any risks and discomfort associated with the experiments, and a signed informed consent form was obtained from each. The protocol for this study was approved by the institutional Ethics Committee.

The inclusion criteria for this study were as follows: healthy volunteers, above the age of 45 years, without signs or symptoms of chronic HF who presented a normal electrocardiography (ECG), and normal pulmonary function at rest or during exercise. Patients were eligible if they had a history of chronic HF secondary to ischemia, alcoholism, hypertensive cardiomyopathy or idiopathic for at least six months, chronic exertional dyspnea despite medical treatment, and a left ventricular (LV) ejection fraction <45% as measured by echocardiography. The exclusion criteria included peripheral vascular disease, significant valvular heart disease (grade > II), uncontrolled hypertension, history of ventricular tachycardia/fibrillation, presence of an implanted cardioverter‐defibrillator, pulmonary disease, or orthopedic comorbidities.

### Study design

This was a transversal study performed in three visits. In the first two visits, subjects performed baseline evaluation: maximal inspiratory pressure (PI_max_) assessment, symptom limited incremental cardiopulmonary exercise testing, spirometry and a knee‐extensor 1 repetition maximum (1RM) test. During this period, both patients and controls also participated in familiarization sessions for the knee extension exercise protocol. In the third visit, the experimental protocol was applied.

### Experimental protocol

**Figure 1.**
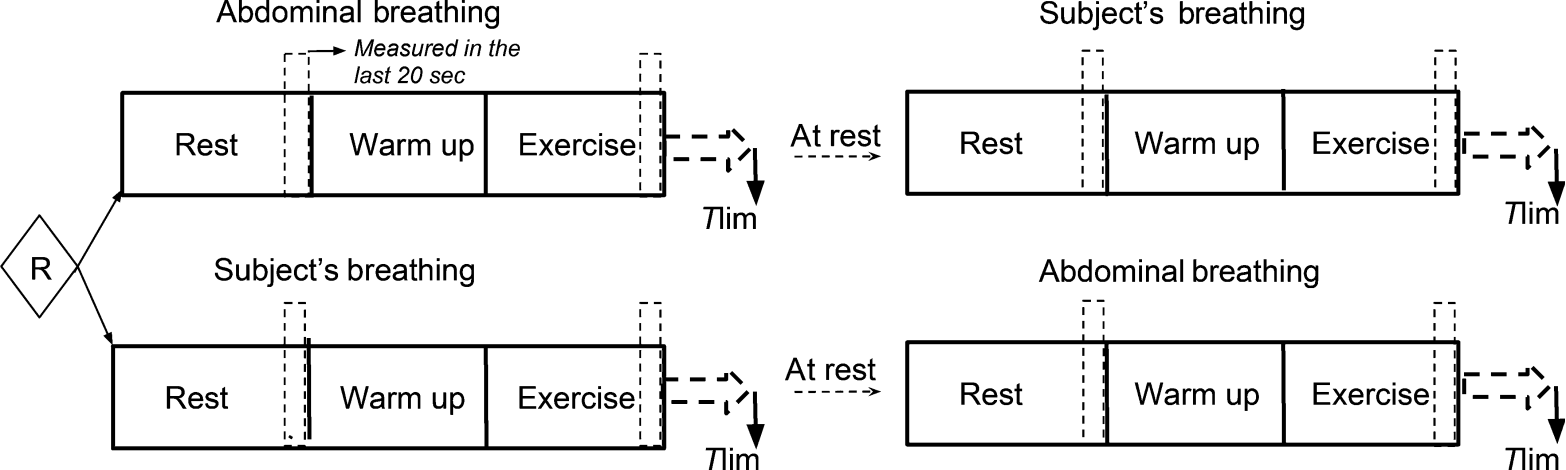
Flowchart of the experimental protocol Dotted squares indicating the moment of the physiological measurements. Subjects were instructed to continue until exhaustion.

The experimental protocol was designed to evaluate the effects of breathing pattern (subject's breathing vs. abdominal) on main outcomes (femoral venous blood return and central hemodynamics) during rest and knee‐extension exercise. Subjects were randomly selected to initiate with one breathing pattern (Phase 1) and, after a 40 min resting interval, repeated the sequence with the other breathing pattern (Phase 2). Each phase includes three periods: rest, warm‐up, and dynamic exercise (knee extension exercise performed with 15% of 1‐RM) (Fig. 1). The first two periods had 5 min of duration each, while the last period, subjects were instructed to continue exercise until exhaustion (time to the limit of tolerance; Tlim). At this moment, dyspnea and leg effort perception were evaluated by the modified categorical Borg scale (Borg 1982).

During the entire protocol, subjects maintained a seated position on an adjustable chair (Acadmix Executive, Metalmix, São Paulo, Brazil) with their knees flexed at 90°. Subjects performed bilateral knee‐extensor exercises with a frequency of 30 min^−1^ and a duty cycle of 0.50, thereby facilitating synchronization of exhale breathing with the knee‐extensor phase and inhale breathing with the relax phase of the lower limb movement.

### Breathing pattern

In the abdominal breathing pattern, subjects were instructed to inhale so that the diaphragm descended and forced an outward excursion of the abdominal wall during inspiration, thereby facilitating a concomitant inspiratory increase in gastric pressure ≥ 6 cmH_2_O. During the subject's breathing, subjects performed their own breathing pattern. Breathing frequency was set at 15 breaths per min ^−1^ with a *T*_I_ / *T*_TOT_ (inspiratory time/total breath time) = 0.50 during both breathing patterns. Changes in gastric (*P*_GA_) and esophageal (*P*_ES_) pressure over the course of a breath were monitored closely throughout the study to ensure that the pressure waveform was uniform.

### Measurements

#### Resting lung function testing

Forced vital capacity (FVC) and forced expiratory volume in 1 sec (FEV_1_) were obtained using a computerized spirometer (Oxycom Delta, Jaeger, Wurzburg, Germany), as recommended by the American Thoracic Society (Miller et al. 2005b).

*P*i_max_ was obtained using a pressure transducer (MVD‐500 V.1.1 Microhard System, Globalmed, Porto Alegre, Brazil), as previously described (Dall'Ago et al. 2006).

The 1RM was performed in the knee‐extensor exercise chair, as previously described by Kraemer et al. (2002).

#### Cardiopulmonary exercise testing (CPET)

Incremental cardiopulmonary exercise test was performed on a treadmill (Inbramed 10200, Porto Alegre, Brazil) (Dall'Ago et al. 2006). Twelve‐lead ECG tracings were obtained during the exercise test (Nihon Khoden Corp., Tokyo, Japan), and blood pressure was measured every 2 min using a standard cuff sphygmomanometer. During CPET and experimental protocol, ventilatory and metabolic parameters were continuously monitored breath‐by‐breath (Oxycom Delta, Jaeger, Wurzburg, Germany).

#### Esophageal (P_ES_) and Gastric (P_GA_) pressure

*P*_ES_ and *P*_GA_ were assessed using thin‐walled balloon catheters (Ackrad Laboratories, Crandford, NJ) coupled to differential pressure transducers. Esophageal and gastric balloons were inserted in the nasal passage and were positioned in the lower one‐third of the esophagus and in the middle‐third of the stomach, respectively. The validity of the esophageal balloon measurements in the subjects was tested using the occlusion method (Baydur et al. 1982). Five to ten maximal sniff maneuvers were performed at the beginning of experimental protocol, and the highest numerical pressure was noted. *P*_ES_ and *P*_GA_ were digitized at 100 Hz using a 10bits analog‐to‐digital converter and displayed in real time on a computer screen. Transdiaphragmatic pressure (*P*_DI_) was determined by calculating the difference between *P*_ES_ and *P*_GA_.

#### Femoral blood flow

Femoral venous blood velocity was measured in the femoral vein, proximal to the vena profunda and distal to the saphenous vein, using an ultrasound Doppler system (En Visor C, Philips, Bothell, WA) (Miller et al. 2005a). The arterial blood velocity was measured in the superficial femoral artery using the same ultrasound Doppler system. Venous and arterial vessel diameter (*d*) was acquired and cross‐sectional areas were calculated using *π* [3.141] (*d*/2)^2^, from the longitudinal vessel image at the point of peak blood velocity. Instantaneous arterial (*Q*_fa_) and venous blood flow (Q_fv_) were calculated using the product of blood velocity and cross‐sectional area achieved at the end of the course of a breath, more precisely at 10 ms after expiration peak.

#### Central hemodynamic

Variables of central hemodynamic were measured noninvasively throughout the experimental protocol using an impedance cardiography device (PhysioFlow PF enduro, Manatec Biomedical, France). The PhysioFlow device and its methodology have been described elsewhere (Borghi‐Silva et al. 2008). Prior to each measurement, the system was autocalibrated by taking into account: age, stature, body mass, and blood pressure values. Verification of the correct signal quality was performed by visualizing ECG tracing, its first derivative (*d*ECG/*dt*), and the impedance waveform (ÄZ) with its first derivative (*d*Z/*d*t) (Charloux et al. 2000).

### Statistical analyses

Data are presented as mean ± standard error of mean. Baseline data for the groups were compared using Student's *t*‐test for independent samples. A generalized estimated equation was used to compare means within groups for each moment of the protocol (at rest and exercise during each breathing pattern). Data were analyzed using the Statistical Package for Social Sciences (version 20 for Windows; SPSS, Inc., Chicago, IL).

## Results

### Participants’ characteristics

Eight healthy controls and nine chronic HF patients were analyzed in this study. Baseline characteristics and patient medications are reported in Table 1.

**Table 1.** Characteristics of study participants

Characteristics	Healthy subjects (*n* = 8)	HF group (*n* = 9)
Demographic/anthropometric		
Age, y	57.4 ± 9.4	60.4 ± 6.7
Weight, kg	81.2 ± 10.7	74.9 ± 14.9
Height, cm	174.3 ± 0.1	167.8 ± 0.07
BMI, kg m^−3^	26.3 ± 1.9	26.9 ± 4.1
RR, breaths per minute	14 ± 2	17 ± 3
HR, bpm	67 ± 6	71 ± 21
SBP, mm Hg	131 ± 10	118 ± 27
DBP, mm Hg	78 ± 6	75 ± 14
LVEF, %		33 ± 9
NYHA Class		I = 3 II = 6
Weber Class		A = 8 B = 1
Etiology (hypertensive/alcohol/ischem/idiop)		1 / 2 / 5 / 1
Comorbidities (HSP/hepatitis A/ DM II)		3 / 1 / 4
Smoking history (no/yes)	6/2	2/7
Pulmonary function		
FEV_1_, L	3.24 ± 0.4	2.0 ± 0.5
FEV_1_, % predicted	90.0 ± 13.1	85.0 ± 14.7
FVC, L	4.2 ± 0.7	2.8 ± 0.4
FVC, % predicted	104.2 ± 11.7	75.8 ± 15.9
FEV_1_ / FVC	0.77 ± 3.9	0.71 ± 15.6
FEV_1_ / FVC, % predicted	97.9 ± 4.9	90.3 ± 19.4
Peak exercise data		
*V*O_2peak_, mL·min^−1^·kg^−1^	36.0 ± 6.0	24.7 ± 9.0
RER	1.19 ± 0.09	1.15 ± 0.07
VE/V_CO2_ slope	21.0 ± 11.3	36.6 ± 4.4
Muscle strength		
*P*i_max_, cm H_2_O	114.1 ± 24.1	93.2 ± 16.9
*P*i_max_, % predicted	106.4 ± 18.7	86.0 ± 14.7
*P*E_max_, cm H_2_O	148.4 ± 20.4	105.5 ± 44.2
*P*E_max_, % predicted	126.1 ± 19.6	93.4 ± 39.9
Sn*P*_DI_, cm H_2_O	105 ± 33	71 ± 22
Sn*P*_ES_, cm H_2_O	−68 ± 23	−56 ± 14
Knee extensor, Load 1RM, kg	97.6 ± 19.3	48.5 ± 10.8
Medication^1^		% Using
Digoxin		66.70
Diuretics		88.90
Long‐acting nitrates		33.30
Statins		66.70
Aspirin		44.40
Beta‐blockers		100
ACE inhibitors		88.90

BMI, body mass index; RR, respiratory rate; HR, heart rate; SBP, systolic blood pressure; DBP, diastolic blood pressure; LVEF, left ventricular ejection fraction; NYHA, New York Heart Association Functional Classification; HSP, high systolic pressure; FEV, forced expiratory volume in 1 sec; FVC, forced vital capacity; *V*O_2peak_, peak oxygen uptake; RER, respiratory exchange ratio; VE, minute volume; Sn*P*_DI_, sniff transdiaphragmatic pressure; Sn*P*_*ES*_, sniff esophageal; 1RM, 1 repetition maximal; ACE inhibitors, angiotensin‐converting‐enzyme inhibitors.

^1^ % in use.

### Respiratory mechanics

Both healthy controls and HF patients were able to understand the instructions and achieved a satisfactory abdominal breathing pattern. The variation in *P*_GA_ (∆*P*_GA_) obtained during abdominal and the subject's breathing pattern were, respectively: 13.0 ± 1.7 vs. 5.0 ± 0.6 (*P *< 0.05) for controls; and 13.8 ± 2.5 vs. 5.5 ± 0.7 cmH_2_O (*P *< 0.05) for patients. Intrathoracic pressures were not significantly different comparing abdominal and the subject's own breathing in both groups (controls: −5.9 ± 1.8 vs. −2.6 ± 0.5 cmH_2_O; HF patients: −5.7 ± 3.7 vs. −3.6 ± 1.7 cmH_2_O; *P *> 0.05).

Both groups of patients also increased satisfactorily their gastric pressures (∆*P*_GA_) using abdominal breathing pattern during exercise (Controls: 10 ± 2.6 vs. 5.9 ± 1.1 cmH_2_O, respectively; *P* = 0.06; HF patients = 11.5 ± 2.6 cm H_2_O and 3.7 ± 1.0 cmH_2_O, respectively; *P *< 0.05). For ∆*P*_ES_ and ∆*P*_DI_, there was no significant difference between breathing pattern in both groups.

### Ventilatory, metabolic and circulatory parameters during experimental protocol

Ventilatory, metabolic and circulatory parameters according breathing pattern at rest and during knee extension exercise are shown in Table 2.

**Table 2.** Ventilatory, metabolic and circulatory parameters according breathing pattern at rest and during knee extension exercise

Parameter measured	At rest		Exercise	
	Subject's breathing	Abdominal breathing	Subject's breathing	Abdominal breathing
*V*T (L)				
Controls	1.3 ± 0.2	1.5 ± 0.1*	1.5 ± 0.2	1.9 ± 0.2*
HF group	1.1 ± 0.1	1.4 ± 0.2*	1.2 ± 0.2	1.6 ± 0.2*
BF (1 min^−1^)				
Controls	17 ± 2.0	16 ± 0.9	18 ± 0.0	16 ± 0.0
HF group	14 ± 0.8	14 ± 0.9	18 ± 1.3	18 ± 2.6
*V*’E (L min^−1^)				
Controls	21.4 ± 5.9	26.0 ± 3.0	31.9 ± 4.7	37.1 ± 5.8*
HF group	14.7 ± 1.1	20.0 ± 2.4*	23.0 ± 2.7	26.8 ± 2.3*
*P*ET_O2_ (mm Hg)				
Controls	122.7 ± 4.2	131.7 ± 2.8*	119.0 ± 4.0	121.1 ± 2.8*
HF group	123.3 ± 2.9	128.0 ± 3.8*	121.3 ± 3.1	121.3 ± 3.4
*P*ET_CO2_ (mm Hg)				
Controls	24.4 ± 2.6	19.0 ± 1.7*	26.4 ± 2.6	24.4 ± 2.1
HF group	24.7 ± 1.9	21.6 ± 2.4*	24.4 ± 2.2	24.1 ± 2.2
*V*O_2_·kg^−1^ (mL·min^−1^·kg^−1^)				
Controls	4.5 ± 0.8	4.6 ± 0.6	7.7 ± 0.6	9.1 ± 0.5*
HF group	3.7 ± 0.3	3.6 ± 0.3	6.3 ± 0.5	7.3 ± 0.5*
Mean blood pressure (mmHg)				
Controls	97 ± 4	96 ± 2	93 ± 5	98 ± 1
HF group	88 ± 5	86 ± 5	93 ± 7	89 ± 5
Heart rate (1 min^−1^)				
Controls	80 ± 6	83 ± 7	98 ± 7	96 ± 4
HF group	76 ± 5	79 ± 5	82 ± 6	81 ± 6

*V*T, volume tidal; BF, breathing rate; *V*’E, minute ventilation; *P*ETO_2_, end‐tidal oxygen tension; *P*ET_CO2_, end‐tidal carbon dioxide tension; *V*O_2_·kg^−1^, oxygen uptake in milliliters of oxygen per kilogram of bodyweight per minute; *V*O_2_, absolute oxygen uptake; METS, metabolic equivalent.

* *P* ≤ 0.05 between subject's breathing and abdominal breathing.

### Effects of breathing patterns at rest

#### Femoral arterial and venous blood flow and central hemodynamic

**Figure 2.**
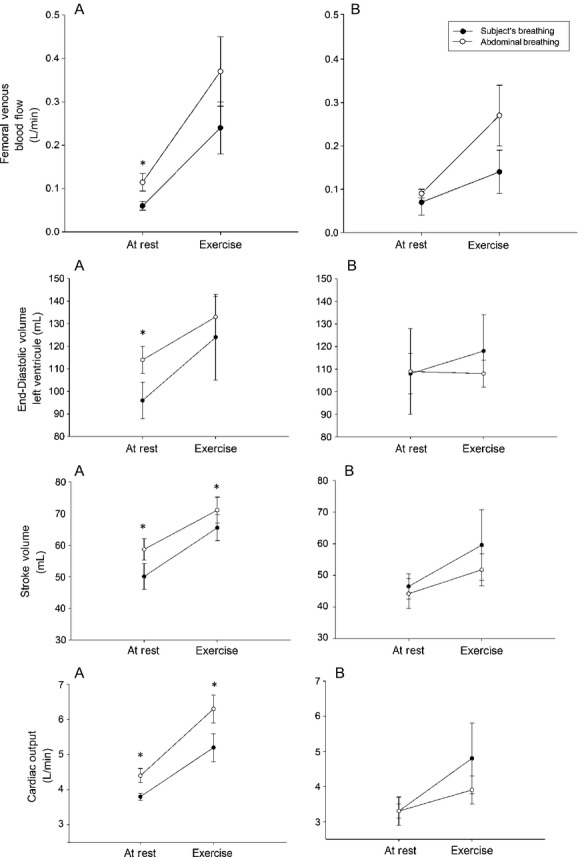
Femoral venous blood flow and central hemodynamic parameters for breathing patterns at rest and during knee extension exercise in healthy controls (A) and HF patients group (B). Data presented in media and standard error. *P ≤ 0.05 between the subject's breathing and abdominal breathing.

For the healthy controls, *Q*_fa_ was not affected according to the breathing pattern employed during a 5 min period (data not shown). However, mean *Q*_fv_, end‐diastolic volume (EDV) of the left ventricle, SV, and cardiac output (CO) were significantly greater during abdominal when compared to the subject's breathing pattern (*P* ≤ 0.05) (Fig. 2).

In HF patients, *Q*_fv_ also increased with abdominal compared to the subject's breathing pattern; however, the change was lower and not statistically significant. Moreover, unlike the control group, this did not influence central hemodynamic parameters (Fig. 2).

**Figure 3.**
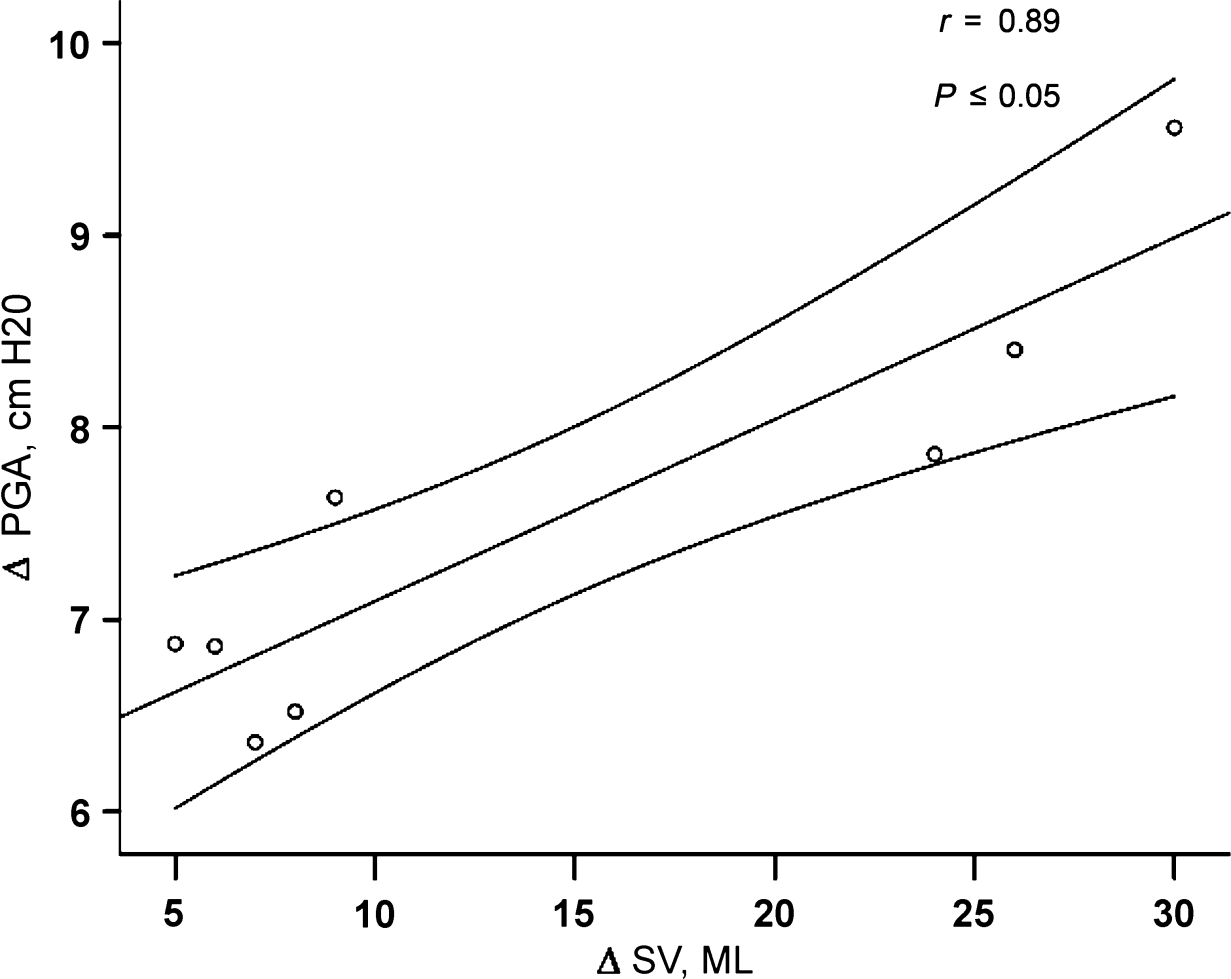
Pearson correlation between the differences in gastric pressure (PGA) and stroke volume (SV) at rest in the healthy controls group.

A significant correlation was found between the increase in SV (∆ SV) and variation in *P*_GA_ (abdominal – subject's breathing pattern; ∆ *P*_GA_) in the control group (*r* = 0.89, *P* ≤ 0.05, Fig. 3). There was no significant correlation between ∆ SV and ∆ *P*_GA_ in breathing patterns in the HF group.

### Effects of breathing pattern during dynamic exercise

#### Femoral venous blood flow and central hemodynamic

Knee extension exercise increased femoral venous blood flow in both groups. In addition, the adoption of abdominal breathing tended to induce an additional improvement (Fig. 2). However, a significant increase in SV and CO was observed only in controls. Otherwise, patients showed a reduction in these parameters with abdominal breathing.

#### Clinical parameters

Adopting abdominal breathing, healthy subjects tended to increase Tlim and reduce leg effort perception adjusted to Tlim, without observed effects on dyspnea. On the other hand, HF patients significantly decreased Tlim and worsened leg effort perception adjusted to Tlim (Table 3).

**Table 3.** Clinical outcomes according to breathing pattern at peak knee extension exercise

Variable	Subject's breathing	Abdominal breathing	*P* value
Tlim (min)			
Controls	8.6 ± 1.0	9.4 ± 0.6	0.196
HF group	10.6 ± 1.9	6.0 ± 0.7*	0.017
Leg Effort (Borg) per Tlim			
Controls	0.71 ± 0.18	0.44 ± 0.07	0.090
HF group	0.67 ± 0.23	1.51 ± 0.31*	0.040
Dyspnea (Borg) per Tlim			
Controls	0.35 ± 0.13	0.29 ± 0.10	0.636
HF group	0.53 ± 0.16	1.04 ± 0.22	0.080

Tlim, time to the limit of tolerance; HF, heart failure. *P ≤ 0.05 between subject's breathing and abdominal breathing.

## Discussion

The major finding of this study was that healthy subjects were able to increase EDV, SV, and CO adopting abdominal breathing pattern, presumably due to increased net cardiac venous return by the abdominal muscle pump. In the context of HF patients with overloaded heart chambers, this was not observed. Furthermore, the same pattern of findings was observed during light exercise, that is, with the contribution of the peripheral muscle pump.

SV is affected by three main mechanisms: the amount of myocardial fiber stretch at the end of diastole (preload); the resistance that the ventricle must achieve to eject blood (afterload); and the inotropic state of the heart (contractility) (Kemp and Conte 2012). As afterload was unaltered (maintained the same arterial blood pressure) and there was no reason to expect change in contractility due to modifications in breathing pattern, it is reasonable to assume that the slight increase observed in SV and CO was secondary to enhancement in preload. In fact, during abdominal breathing, we observed an increased *Q*_fv_ in the expiratory phase. It is consistent with the concept that abdominal pattern of breath modulation on femoral venous return is reversed compared to nondiaphragmatic breathing; femoral venous return is reduced during the inspiratory phase of a diaphragmatic breath, with resurgence of blood flow during the expiratory phase of the cycle, resulting in no net effect of breathing pattern *per se* on steady‐state femoral venous return. It means that any reduction of venous return during diaphragmatic inspiration is balanced by an equal or opposite resurgence of venous return during ensuing expiration (Miller et al. 2005a). The enhancement in cardiac venous return is explained by the emptying of splanchnic circulation caused by an increase in abdominal pressure. Splanchnic emptying shifts blood to the hepatic vein and increases the blood pressure at its entry into the inferior vena cava (IVC), eliminated the pressure gradient that produces flow between the femoral vein and the IVC. In other words, the circulatory function of the diaphragm produces an oscillatory composition of inferior vena cava blood. During inspiration, splanchnic venous return is favored, whereas, during expiration, venous return of blood below the entry of the hepatic vein is favored (Aliverti et al. 2009, 2010). The consequence is that abdominal breathing promotes an extra blood volume mobilization (from splanchnic circulation) increasing net venous return to IVC, that is, diaphragmatic inspiration implies that inferior vena cava venous return is facilitated primarily by the central translocation of blood from the vessels of the abdomen and is not the result of facilitation of venous return from the lower limbs (Miller et al. 2005a). The final consequence is the increment in EDV and subsequently, via frank‐starling mechanism, in SV and CO (Aliverti et al. 2010).

The above rational is corroborated by the fact that same findings were not observed in patients with HF during abdominal breathing; despite a tendency to increase *Q*_fv_, no significant modification was observed in EDV and SV. LV dysfunction causes an increase in the amount of blood in the ventricle and therefore an increase in both end‐systolic and end‐diastolic volumes and, consequently, pressures. LV failure is the most common cause of right ventricular (RV) failure. As the RV fails, there is an increase in the amount of blood in the ventricle, which leads to elevated right atrial pressure and increased pressure in the vena cava system which impairs venous drainage. This leads to increased pressure in abdominal circulation and extremities reducing the driving pressure for venous return (Kemp and Conte 2012).

We also aimed to investigate the synchronism between abdominal and skeletal muscle during mild dynamic exercise. The rhythmic contraction of the appendicular muscles during exercise results in compression of the intramuscular veins, facilitating the blood venous return to the heart (Stewart et al. 2004). It was previously demonstrated that during calf contraction, the modulation of abdominal breathing on limb venous return was maintained (Miller et al. 2005a). The synchronization of peripheral muscles with abdominal muscle pump would improve venous return during expiration. However, quadriceps contraction during expiration (period when the compensatory resurgence of the flow occurs from the blood pooled in the lower limbs from the previous inspiration) invalidates the statistical significance observed in hemodynamic parameters with abdominal breathing. This assistance (peripheral muscle pump forcing blood centrally) partially balances both breathing patterns regarding *Q*fv. Nonetheless, a nonstatistically significant increase in *Q*fv and EDV could be observed in healthy subjects, resulting in a statistically significant increase in SV and CO. It means that even with the addition of quadriceps pump in healthy controls, the physiological benefits, in terms of venous return and central hemodynamic, were maintained. Again, during exercise, as observed at rest, abdominal breathing caused lower values of SV and CO in HF patients. Additionally, exercise duration until the limit of tolerance was reached, tended to increase in healthy subjects with abdominal breathing, without an increase in leg effort and dyspnea perception adjusted to the exercise time. On the other hand, HF patients that did not present favorable physiological effects with abdominal breathing, showed a significant lower exercise tolerance and higher limb discomfort/Tlim and dyspnea/Tlim ratio with abdominal breathing pattern (Table 3). It supports the idea that abdominal breathing could be a clinically beneficial strategy during exercise training in subjects without cardiac overloaded chambers. However, abdominal breathing should not be recommended for HF patients.

Although subjects were encouraged to maintain similar tidal volumes across each breathing pattern, both groups slightly increased minute ventilation and reduced *P*ET_CO2_. This could affect arterial inflow to lower limbs and consequently their venous return via competition between ventilatory and peripheral muscles (Harms et al. 1997) or vasomotor effects of hypocapnia(Chin et al. 2010). Nevertheless, the expected effects of these mechanisms would be reduction in *Q*fa. We found no effect in *Q*fa contrasting each breathing pattern.

The present study demonstrates that in healthy controls, abdominal pressure produced by inspiratory muscles can increase net cardiac venous return and improve SV and CO. This same pattern was observed during exercise. In HF patients, these findings were not observed, mostly likely due to the fact that elevated pressures in the atrium and vena cava system compromised venous drainage from the body.

## Conflict of Interest

No conflict of interests were observed by the authors.
